# The Role of Mitochondrial Quality Control in Cardiac Ischemia/Reperfusion Injury

**DOI:** 10.1155/2021/5543452

**Published:** 2021-06-09

**Authors:** Jia Huang, Ruibing Li, Chengbin Wang

**Affiliations:** ^1^Department of Clinical Laboratory Medicine, the First Medical Centre, Chinese PLA General Hospital, China; ^2^Medical School of Chinese PLA, Beijing, China

## Abstract

A healthy mitochondrial network produces a large amount of ATP and biosynthetic intermediates to provide sufficient energy for myocardium and maintain normal cell metabolism. Mitochondria form a dynamic and interconnected network involved in various cellular metabolic signaling pathways. As mitochondria are damaged, controlling mitochondrial quantity and quality is activated by changing their morphology and tube network structure, mitophagy, and biogenesis to replenish a healthy mitochondrial network to preserve cell function. There is no doubt that mitochondrial dysfunction has become a key factor in many diseases. Ischemia/reperfusion (IR) injury is a pathological manifestation of various heart diseases. Cardiac ischemia causes temporary tissue and organelle damage. Although reperfusion is essential to compensate for nutrient deficiency, blood flow restoration inconsequently further kills the previously ischemic cardiomyocytes. To date, dysfunctional mitochondria and disturbed mitochondrial quality control have been identified as critical IR injury mechanisms. Many researchers have detected abnormal mitochondrial morphology and mitophagy, as well as aberrant levels and activity of mitochondrial biogenesis factors in the IR injury model. Although mitochondrial damage is well-known in myocardial IR injury, the causal relationship between abnormal mitochondrial quality control and IR injury has not been established. This review briefly describes the molecular mechanisms of mitochondrial quality control, summarizes our current understanding of the complex role of mitochondrial quality control in IR injury, and finally speculates on the possibility of targeted control of mitochondria and the methods available to mitigate IR injury.

## 1. Introduction

Mitochondria are the primary sites where eukaryotic cells conduct aerobic respiration. The myocardium highly relies on aerobic metabolism to maintain its cellular viability and systolic function. Mitochondria account for over 30% of the total volume of cardiomyocytes in the myocardium to produce sufficient ATP and are closely related to cardiomyocyte metabolism [1]. Therefore, the self-regulation mechanism to maintain the normal function of mitochondria is fundamental. Mitochondria undergo specific physiological processes related to quantity, shape, and quality in response to physiological environment changes to ensure cardiomyocyte activity. These physiological processes are prerequisites for mitochondrial quality control, including mitochondrial biogenesis, protein homeostasis maintenance, mitochondrial fission/fusion, and removal of damaged mitochondria or protein by fusing with lysosomes [2, 3]. The mitochondrial fission and fusion processes segregate damaged mitochondria and promote the balance of mitochondrial components such as DNA and proteins. Dysfunctional mitochondria are cleared and recycled by lysosomes under oxidative stress and nutrient deprivation in a mitophagic fashion to form mitochondrial spheroids or mitochondrial-derived vesicles (MDVs). Mitochondrial biogenesis is responsible for a healthy mitochondrial network via mitochondrial turnover in cardiomyocytes [3–5].

Meanwhile, mitochondria are also closely linked to cell death in both necrotic and apoptotic forms. Under ischemia/reperfusion (IR) injury, mitochondria are vulnerable to cell stress such as hypoxia and oxidative stress built-up by ischemia and reperfusion, leading to overproduction of reactive oxygen species (ROS), Ca^2+^ overload, and apoptotic proteins activity. This will open the mitochondrial permeability transition pore (mPTP) and form a channel to release cytochrome c into the cytoplasm, inducing a cascade of apoptosis [6]. When exposed to mitochondrial injury, cells first respond to active antioxidation, repair DNA, and regulate protein folding to maintain original structure and composition. If the first line of defense fails, the quality control system will be activated. Changing the mitochondrial function and structure is not only an adaptive response to ischemia and reperfusion but also a key process of apoptosis or necrosis of myocardial cells. Therefore, targeted intervention in mitochondrial quality control may slow down the degree of IR damage to some extent.

## 2. Mitochondrial-Centric Damage in Ischemia/Reperfusion Injury

Ischemia-induced tissue damage is a major cause of fatal disease. IR injury is a leading cause of chronic heart failure and the main pathological manifestation of coronary artery disease (CAD) [7]. Acute myocardial infarction (AMI) is induced by coronary artery occlusion, causing a cessation of blood flow (ischemia) and reperfusion damage [8]. Myocardial ischemia injury is mainly caused by myocardial hypoxia and nutrient deprivation resulting in necrosis or temporary functional impairment of myocardial cells. The free radicals produced by reoxygenation after long-term ischemia are the real factors that cause tissue damage. ROS were previously believed to kill cells directly through oxidative stress. However, the current view gradually favors that ROS triggers physiological or procedural pathways of cell death [9], for example, by inducing prolonged mPTP opening, which ultimately destroys mitochondria, cells, and tissues. Among various mechanisms speculated for cardiac IR injury, such as oxidative stress, mitochondrial Ca^2+^ overload, endothelial dysfunction, inflammation, autophagy failure, and apoptosis, mitochondria play a central role in mediating these pathophysiological processes with impaired mitochondrial function [10, 11]. The prolonged mPTP opening is the key link to tissue damage caused by mitochondria. Due to the existence of voltage-dependent anion channels (VDAC), the outer mitochondrial membrane (OMM) is much more permeable than the inner mitochondrial membrane (IMM), allowing metabolites and certain small molecules to exchange between cytoplasm and mitochondria. The permeability of IMM is determined by mPTP [12]. Transient mPTP opening has a critical physiologic role in regulating ROS signaling, cardiomyocyte development, and mitochondrial Ca^2+^ outflow. Prolonged mPTP opening results in depolarization of mitochondrial membrane potential, ATP synthesis cessation, and mitochondrial swelling and death [13]. Unregulated opening of mPTP is a key factor in inducing ischemia-reperfusion injury and heart failure [14]. Therefore, stringent mitochondrial quality control is critical during all IR injury stages.

After ischemia and reperfusion, microcirculation disturbance and tissue damage gradually develop, including ischemia, acute, subacute, and chronic reperfusion stages [15]. At the ischemia stage, hypoxia and nutritional deficiencies block subunits of mitochondrial respiratory chain expression, decreasing ATP synthesis [16]. Coupled with ATP consumption by surrounding tissues, ATP amount is hard to meet the energy demands for actin polymerization to form contractile devices in heart muscle cells [17]. Additionally, ATP deprivation can also lead to endothelial junction protein phosphorylation and increased vascular permeability [18]. Reperfusion is undoubtedly crucial for restoring blood supply and myocardial salvage. The current clinical practice believes that early rapid patency, a short time to reperfusion, and complete restoration of normal flow can effectively reduce overall mortality [19]. However, this process will also cause further damage, such as continuous ATP decline and excessive peroxide generation from mitochondria, even excess insult during initial ischemia. Injury and downregulation of complex I and II in mitochondria block ATP production. Moreover, electron transfer during reperfusion also causes complex I to form large amounts of peroxides and ROS release [20]. Elevated mitochondrial ROS levels not only drive mitochondrial oxidative damage and disturbing respiratory mechanism and ATP production but also attack cellular components and promote releasing inflammatory cytokines through activating several intracellular signaling pathways [21]. In intact cardiomyocytes, mitochondrial ROS and calcium dysregulation result in prolonged mPTP opening, providing a channel for releasing cytochrome c, activating classical mitochondrial-death pathway by acting with caspase-9 and caspase-3 in cytoplasm [22, 23]. In addition, mPTP not only opens to change mitochondrial membrane potential and induces mitochondrial-dependent apoptosis under long-term ischemia and reoxygenation but also further disrupts the respiratory chain and simultaneously produces more ROS, leading to IR-induced apoptosis and necrotic cell death, which is called ROS-induced ROS release (RIRR) [12]. The RIRR then spreads and amplifies damage to other tissues [24], while ATP absence and oxidative stress during reperfusion will further stimulate mPTP to aggravate the damage.

Furthermore, mPTP opening was also closely correlated with increasing mitochondrial matrix Ca^2+^ ([Ca^2+^]_m_). Intracellular Ca^2+^ ([Ca^2+^]_i_) enters the mitochondrial matrix through a group of highly selective Ca^2+^ channels in IMM called mitochondrial calcium uniporters (MCUs) and stimulates ATP production under physiological conditions [25]. However, excessive Ca^2+^ entering mitochondria increases [Ca^2+^]_m_ levels that may activate mPTP and harm mitochondrial function. The current view supports that [Ca^2+^]_i_ and [Ca^2+^]_m_ are involved in mitochondrial quality control and regulate appropriate mitochondrial function, through which specific proteins are produced and eliminated during normal physiological functions and mitochondrial and endoplasmic reticulum stress [26]. In some genetic studies, MCU deficiency seems to alleviate cardiac IR injury, suggesting that there may be less Ca^2+^ entering mitochondria via MCU in these models, but this hypothesis has not been quantitatively tested [27, 28].

**Figure 1:**
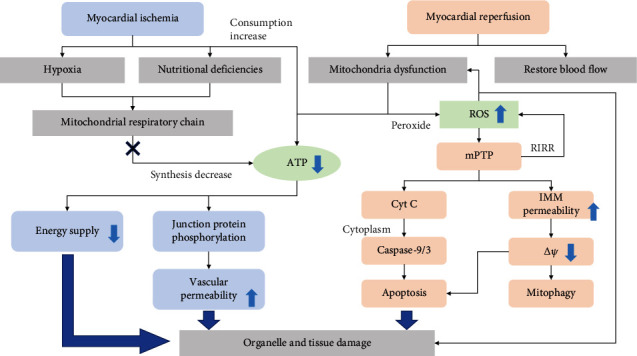
Injury mechanism involved in mitochondria at different stages of myocardial ischemia-reperfusion. Ischemia/reperfusion (IR) injury is divided into two stages, ischemia and reperfusion. Both involve a decline in ATP synthesis. In the phase of ischemia, the damaged mitochondrial respiratory chain will reduce ATP synthesis, coupled with continuous energy consumption of other tissues and organelles, resulting in a significant decrease in ATP content. Due to a lack of energy supply and increased vascular permeability, myocardial ischemia can cause temporary tissue damage. In the reperfusion phase, in addition to the continuous decline of ATP synthesis, the mitochondrial respiratory chain will also excessively produce ROS. ROS mediates prolonged mPTP opening that forms a channel to release cytochrome c and then activate the apoptotic cascade of cardiomyocytes, which further aggravates tissue damage.

The central role of mitochondria in cardiac IR injury has been well proven, but the causal relationship and potential mechanism during cardiac IR injury of mitochondrial quality control remain unexplored. This article reviews the mechanism of mitochondrial quality control and its role in ischemia-reperfusion injury (Figure 1).

## 3. Mitochondrial Dynamics

### 3.1. Mitochondrial Fission

Mitochondria are highly dynamic in the cardiovascular system and are spatially and functionally organized in a filamentous network undergoing fusion and fission, through which mitochondria constantly change between elongated and fragmented morphology responding to various environmental stimuli and cellular requirements [29, 30]. In mammalian cells, mitochondrial fission is mediated by dynamin-related peptide 1 (Drp1), mitochondrial fission protein 1 (Fis1), mitochondrial fission factor (Mff), and 49kd and 51kd mitochondrial dynamics proteins (Mid49/51), while mitochondrial fusion is primarily regulated by dynamin-related GTPases mitofusins (Mfn1 and Mfn2) and optic atrophy protein 1 (Opa1) [31]. Drp1 distributed widely in cytosol and translocases to OMM when activated by phosphorylation/dephosphorylation via actin and microtubule mechanisms [32]. After that, Drp1 interacts with Fis1, Mff, and Mid49/51 and then constricts and cleaves mitochondria by GTP hydrolysis [33]. As a key factor in mediating fission, regulating Drp1 at multiple levels, including transcriptional control, alternative splicing, and posttranslational modification is important [34]. In immune cells, Drp1-X01 subtype, unique to microtubules and phosphorylated for fission, is derived from alternative splicing of Drp1. This splicing variant stabilized microtubules and resulted in reduced apoptosis [35]. However, major regulatory mechanisms in most cardiovascular diseases are posttranslational modification processes, including phosphorylation, dephosphorylation, ubiquitination, sumoylation, nitrosylation, and acylation [34, 36]. Protein kinase A (PKA) phosphorylates and inactivates Drp1 while Ca^2+^–calmodulin-dependent phosphatase calcineurin dephosphorylates Drp1 and promotes mitochondrial fission [37, 38]. Drp1 phosphorylation at Ser616 promotes its oligomerization around OMM and induces division loop formation at the mitochondrial potential fission site [39]. In contrast, phosphorylation at Ser637 inhibits Drp1 oligomerization and prevents mitochondrial division [40].

Many studies focus on distinct functions of Drp1-mediated fission involved in diverse biological processes. On the one hand, mitochondrial fission is regarded as a prerequisite for mitophagy through which dysfunctional mitochondria containing damaged proteins, destabilized membranes, and mutated or damaged mitochondrial DNA (mtDNA) are segregated [41]. Additionally, Drp1-serine 616 phosphorylation allows the mitochondria to be evenly distributed in daughter cells during mitosis [40]. However, in response to IR injury, on the other hand, Brady et al. [42] first discovered extensively fragmented mitochondria when Bax translocates from cytosol to mitochondria during ischemia, which could be disturbed by mPTP CsA and p38 MAPK SB203580 inhibitors. Moreover, Karbowski et al. [43] inhibited apoptotic fragmentation of mitochondria with Drp1K38A, a dominant-negative mutant of Drp1, and found that Bax translocation to potential mitochondrial scission sites cannot be affected, improving that Bax initiates apoptotic fragmentation through Drp1 mediation. Moreover, Mff-mediated fission, a receptor of Drp1, was also reported to hold essential function in fatal mitochondrial fission during IR injury [44, 45]. Acute cardiac IR injury upregulates NR4A1 expression, nuclear receptor subfamily 4 group A member 1, to activate serine/threonine kinase casein kinase2 *α* (CK2*α*), which phosphorylates and activates Mff, enhancing Drp1 translocation and producing detrimental fragmented mitochondria [44]. Mff binds to Drp1 to induce mitochondrial division, leading to excessive ROS production and oxidizing cardiolipin. It also triggers hexokinase 2 (HK2) dissociation, opens mPTP, releases mitochondrial cytochrome c into cytoplasm, and initiates caspase-dependent apoptosis [46]. DUSP1, dual-specificity protein phosphatase1, is downregulated in cardiac IR injury to promote the phosphorylation level of Mff via JNK pathway activation. DUSP1 restoration could alleviate the lethal mitochondrial division and promote cell survival in myocardial tissue [45].

### 3.2. Mitochondrial Fusion

Compared to mitochondrial fission, mitochondrial fusion is usually crucial for the health and physiological functions of mitochondria, including replenishing damaged mitochondrial DNAs and maintaining membrane potential [47]. Mfns were first discovered in 2001 embedded in OMM fusing adjacent mitochondria through concerted oligomerization and GTP hydrolysis, while Opa1 is situated in IMM and mediates its fusion [48]. Ablation of Mfn1 and Mfn2 genes in adult mice (8 weeks of age) hearts resulted in mitochondrial rupture, impaired mitochondrial respiratory function, and severe fatal cardiomyopathy after 7-8 weeks, suggesting that mitochondrial fusion proteins are essential for normal myocardial mitochondrial morphology and respiratory function [49]. Studies indicate that Mfn1 plays different roles from Mfn2. Mfn1 is primarily responsible for the fusion of two mitochondria, whereas Mfn2 acts as a protein stabilizing the interaction because of lower GTPase activity [50]. In cardiac IR injury, overexpression of Mfns and Drp1 inhibition prevents mPTP opening, which is a critical mediator of IR injury and reduces cell death [51, 52]. Ablation of Mfns or Opa1 induces mitochondrial fragmentation that can activate mitochondrial apoptosis in IR injury [53]. The activity of Mfns is inhibited by ubiquitination and blocking the process of removing damaged mitochondria by mitophagy [54]. In mice, myocardial IR, and myocardial cell hypoxia/reoxygenation models, MCU upregulation responsible for calcium overload, which underlies mPTP opening during the IR phase, increases calpain expression, which is proved to blunt Opa1 expression and activate calcineurin to phosphorylate Drp1 leading to excessive fission [55].

In conclusion, restoration or stimulation of fusion may be an effective means to reduce myocardial IR injury. A study found that melatonin can upregulate OPA1 expression transcriptionally through the AMPK pathway, thereby increasing the ability of mitochondria and cardiomyocytes to survive under IR injury [56]. Pharmacological mitochondrial fusion promoter-M1 could increase Mfn2 expression to regulate the dynamics and reduce mitochondrial dysfunction. Administration of M1 before ischemia can significantly improve reducing mitochondrial fusion protein observed in cardiac IR injury, reduce arrhythmia incidence, and reduce the infarct area and cardiac apoptosis, thus, preserving cardiac function and reducing mortality. M1 given during ischemia and at the beginning of reperfusion also has a cardioprotective effect but is less effective than M1 given before ischemia [57]. Another study reported that M1 also improves brain mitochondrial dysfunction and blood-brain breakdown induced by cardiac IR injury [58]. These findings suggest that these may be promising interventions that offer cardioprotective effects in the clinical setting of myocardial IR injury.

## 4. Mitophagy

Mitophagy is an evolutionarily conserved self-degradation process in which mitochondria are delivered to lysosomes for degradation in a selective macrophage form. Typically, basic mitophagy performs as a life-sustaining mechanism through the circulation of proteins and metabolites, especially under nutrient deprivation. In the heart, the mitophagy level needs to adapt to environmental changes and respond to various heart diseases, such as cardiomyopathy due to ischemia/reperfusion, cardiac hypertrophy, and heart failure [59]. However, how individual mitochondria are identified and whether mitophagy plays a protective or harmful role in heart disease has not yet been determined. In most cases, mitochondria can clear mitochondrial defects in IR injury and are considered a protective or adaptive mechanism. However, uncontrolled or excessive (maladaptive) mitochondria may lead to a shortage of functional or healthy mitochondria produced by ATP, resulting in impaired cell survival.

The current view is that initiating mammalian autophagy is activated by phosphorylation of the ULK1 complex (ATG13, ULK1, and FIP200), a downstream target of AMPK-mTOR and that ATG9 vesicles fuse with lipid membranes derived from the endoplasmic reticulum to form a phagophore [60, 61]. Using Atg12-Atg5-Atg16 and LC3/Atg8 systems, damaged mitochondria are recognized and encapsulated by LC3 on endoplasmic reticulum-derived bilayer structure and eventually degraded by fusing with lysosomes. It is currently known that phagophore mitochondria recognition can be mediated by several pathways and play primary roles under distinct tissues and situations. However, mitochondrial identification pathways are coordinated with each other in the mitophagy process rather than being independent [62]. Nevertheless, specific connections and mechanisms between these independent pathways require further research.

### 4.1. PINK1/Parkin-Mediated Mitophagy

PINK1/Parkin-mediated mitophagy is a ubiquitin-dependent pathway that mainly plays a role in the nervous system. Under normal circumstances, PINK1 is located in IMM and is rapidly degraded by PARL [63]. When mitochondrial membrane potential stability decreases, PINK1 is transferred to OMM, phosphorylating, and recruiting Parkin, or phosphorylated PINK1 directly ubiquitinates other outer membrane proteins [64]. The ubiquitinated outer membrane protein acts as a signal to promote phosphorylation of cargo receptors by kinase TBK1. Cargo receptors contain LC3/GABARAP-interacting region (LIR) motifs, which can recruit LC3 to mitochondria and mediate phagocytosis. Other cargo receptors such as p62, NBR1, and TAX1BP1 have been shown to play an essential role in other selective autophagy, but their role in mitophagy is weak [65]. Due to its unique relationship with Parkinson's disease, PINK1/Parkin-mediated mitophagy pathway has been extensively studied, mainly in brain tissues. However, many recent studies have demonstrated the protective role of the PINK1/Parkin-mediated mitophagy pathway in cardiac IR injury [56]. Yu et al. [66] found that patients with diabetic cardiomyopathy (DCM) have an increased susceptibility to myocardial IR injury. In the DCM model of IR injury, Drp1-mediated mitochondrial fission was enhanced (mean mitochondrial size was significantly reduced, the number of fragmented mitochondria was significantly increased), oxidative phosphorylation complex was damaged, and FUNDC1 and Parkin expressions involved in mitophagy were also significantly decreased. Melatonin reversed this adverse effect through SRIT6 and AMPK-PGC-1*α*-Akt signaling and protects the myocardium from IR injury. It also suggests an inseparable relationship between mitochondrial biogenesis, division, and mitophagy. In contrast, Zhou et al. [67] proposed that IR injury opens mPTP and promotes Parkin-mediated mitophagy and ultimately leads to cell death. This phenomenon has a negligible effect on the signal pathway involved in mitophagy, but due to homeostasis disorder caused by excessive mitophagy, which leads to declining ATP production capacity, causing mitochondria to be unable to meet the basic requirements of cells for energy and reduces the resistance of cells to IR injury.

### 4.2. BNIP3/NIX-Mediated Mitophagy

The second is a receptor-mediated mitophagy pathway, which BNIP3/NIX mediates. BNIP3 (Bcl2 and adenovirus E1B19KDa interacting protein 3) and NIX (BNIP3-like) are proteins homologous to Bcl-2 in BH3 domain and embedded in mitochondria and endoplasmic reticulum. NIX and BNIP3 have putative and classical LIR, respectively. Phosphorylation of serine residues 17 and 24 on both LIR sides of BNIP3 promotes its binding to LC3 [68]. The protein structure determines the dual function of BNIP3/NIX, which induces cell death and participates in mitophagy [69]. Physiologically, the BNIP3 expression level was very low in organs, and NIX is involved in mitophagy promoting degradation of mitochondria in reticulocytes and plays an indispensable role in the maturation process of red blood cells [70]. In cardiomyocytes, the pathological mechanism involved in NIX is mainly related to cardiomyocyte hypertrophy and regulated by G*α*q-dependent signaling, while BNIP3 is significantly induced by hypoxia in addition to a combination of hypoxia and acidosis under prolonged myocardial ischemia [71, 72]. Studies have confirmed that BNIP3 is a downstream target of hypoxia-inducing factor-1*α* (HIF-1*α*). The expression level of BNIP3 is regulated by HIF-1*α* transcription to determine cardiac cell death and mitophagy in the case of hypoxia built-up by IR injury or cancer. In addition to HIF-1, BNIP3 is also the target of other transcription factors, such as PlAGL2, E2F1, and FoxO3, which eventually induce apoptosis and mitophagy [73].

BNIP3 causes cell death in three ways, including activating mitochondrial-dependent apoptosis pathway, inducing cell necrosis, and triggering pyroptosis [74]. Hypoxia-induced cardiomyocyte death demonstrates apoptotic characteristics, although it is unclear whether caspase is involved in this process [72, 75]. BNIP3/NIX interacts with BCL-2 and BCL-XL and induces apoptosis through the C-terminal transmembrane domain, and both have similar promoting activities [76]. Mice with gene ablation or dominant inhibition of BNIP3 can reduce apoptosis of cardiomyocytes in hypoxia and significantly improve ventricular remodeling after IR injury [77]. Hypoxia also upregulates EIF4A3- (eukaryotic translation initiation factor 4A3-) induced Circ-BNIP3 and promotes BNIP3 expression through performing as a miRNA-27a-3p sponge to aggravate hypoxia-induced injury with increased caspase-3 activity and Bax level [78]. Apart from inducing apoptosis, BNIP3 also causes mitochondrial depolarization via mPTP opening and mediates the BNIP3-caspase-3-GSDME pyroptosis pathway in cardiac injury [79]. However, mitophagy function mediated by BNIP3/NIX remains controversial.

When mitochondria are exposed to external stimulation, BNIP3 dimerizes and binds to LC3 to activate mitophagy [80]. In IR injury, hypoxia and ROS activate BINP3-mediated mitophagy by inducing HIF-1 expression and play a protective role in myocardial ischemia reperfusion [81]. In diabetic ischemia-reperfusion model, a combination of deferoxamine (DFO) and sevoflurane posttreatment (SPostC) can protect the myocardium through HIF-1/BNIP3-mediated mitophagy [82]. Another study showed that berberine (BBR) could induce cardiomyocyte proliferation, inhibit cardiomyocyte apoptosis, and enhance HIF-1*α* and BNIP3 promoter binding to mediate BNIP3 expression, thereby activating the HIF-1*α*/BNIP3-mediated mitophagy pathway and protecting myocardial IR injury [83]. However, according to another experiment, Bnip3 expression was upregulated in cells treated with hypoxia and reoxygenation to mimic IR condition in vitro. DUSP1 was downregulated after acute cardiac IR injury and amplified BNIP3 phosphorylation and activation through the JNK pathway, leading to mitophagy which eventually caused myocardial injury [45]. Therefore, BNIP3/NIX exhibited dual properties during myocardial ischemia/reperfusion injury, inducing cell death and participating in mitophagy process. In some cases, BNIP3/NIX-induced mitophagy is defensive, while in others, it leads to cell death. Mitophagy-related cell death is unclear whether it is due to overmitophagy or the process of mitophagy itself is fatal.

### 4.3. FUNDC1-Mediated Mitophagy

Another mitophagy receptor, FUNDC1 (FUN14 domain containing 1), a mitochondrial protein on OMM, has also been confirmed to activate hypoxia-induced mitophagy [84]. FUNDC1 contains three transmembrane (TM) domains. The N-terminal region is exposed to the cytoplasm with a typical LIR, Y (18) XXL. Conservative Y18 and L21 are essential to mediate the interaction between FUNDC1 and LC3 [85]. It is regulated by LIR motif autophosphorylation instead of transcription [86]. Currently, FUNDC1 dephosphorylation is believed to be an activated state, which can promote mitophagy and protect cardiomyocytes and endothelial cells. In contrast, FUNDC1 phosphorylation by upstream factors will inactivate it, hinder mitophagy, and have a damaging effect on the heart [85]. CK2*α* is upregulated after acute myocardial ischemia-reperfusion injury. CK2*α* effectively inhibits mitophagy by phosphorylating and blunting FUNDC1 function through upregulating NR4A1 expression, finally leading to the failure to clear mitochondrial damage and mitochondrial apoptosis [44, 87]. Regarding depolarization of mitochondrial membrane potential or hypoxia, mitochondrial phosphoglycerate mutase PGAM5 dephosphorylates and activates FUNDC1 at ser-13, promoting mitophagy occurrence, which can be reversed by CK2*α* [88]. FUNDC1 as a substrate of Src kinase can be phosphorylated and inactivated at Tyr-18, which will prevent mitophagy. Src kinase inactivation during hypoxia dephosphorylates FUNDC1, and LC3 will preferentially bind to dephosphorylated FUNDC1 and promote mitophagy under hypoxic conditions [85]. Interestingly, ULK expression increases under hypoxia or mitochondrial uncoupling agent (FCCP) and translocates to mitochondria that need to be cleared. FUNDC1, as a substrate of ULK, binds with ULK and is phosphorylated at ser-17, promoting the interaction between FUNDC1 and LC3, which is essential for phagocytic vesicles to recognize damaged mitochondria [86]. The mTORC1-ULK1-FUNDC1 pathway mediates mitophagy, effectively regulates mitochondrial quality and cell survival, and inhibits the occurrence of myocardial ischemia-reperfusion injury [89].

FUNDC1 in cardiomyocytes also binds to ER-resided inositol 1,4,5-trisphosphate type 2 receptor (IP3R2), modulating calcium ions release from ER into mitochondria and cytosol. FUNDC1 ablation accumulates calcium ions in ER and reduces the level of calcium ions in cytoplasm, which suppresses the expression of mitochondrial fission 1 protein (Fis1) to raise elongated mitochondria and mitochondrial dysfunction [90]. ATFS-1 is a transcription factor that plays a central role in the mitochondrial unfolded protein response (UPR^mt^). In hypoxia-reoxygenation, the protective effect of FUNDC1 on cardiomyocytes also needs to be coordinated with ATFS-1. In the absence of FUNDC1, ATFS-1-dependent stress response and metabolic remodeling will occur [91].

In conclusion, FUNDC1-mediated mitophagy plays a prosurvival role in reperfusion heart tissue. Mammalian STE20-like kinase 1 (Mst1) is significantly increased in reperfusion heart, which decreases FUNDC1 expression through MAPK/ERK-CREB pathway. The protective mitophagy loss increases tissue damage in cardiomyocytes [92]. FUNDC1-induced mitophagy was proved to protect the myocardium by inhibiting platelet activation. Physiologically, mitophagy maintains good mitochondrial quality and platelet activation by clearing “toxic” platelet mitochondria. In IR injury, vascular obstruction caused by platelet adhesion constitutes a hypoxic environment that increases FUNDC1-mediated mitophagy level in platelets and subsequently reduces platelet activation to prevent I/R injury deterioration [93]. Therefore, it seems that we can further explore the FUNDC1 domain and explore more sites that can be regulated by phosphorylation or dephosphorylation after transcription or translation, as well as factors or proteins that target these sites. The IR injury degree could be regulated by intervening in the mitophagy pathway involved in FUNDC1.

## 5. Mitochondrial Biogenesis and Proteostasis

Given the role of mitochondria in energy production, they are usually exposed to peroxide like ROS leading to mitochondrial DNA (mtDNA) mutations and protein misfolding. Mitophagy clears damaged ones, and mitochondrial biogenesis generates new mitochondrial ingredients, including protein and lipids, which cooperate to ensure reticular mitochondria replenishment. Mitochondrial biogenesis can be regarded as the growth and division of the original mitochondria. Three parts, including mitochondrial genome, proteins, and lipids on IMM and OMM, ensure the complete biological function of mitochondria and spatial structure of biochemical reaction [94]. Human mtDNA is a double-stranded loop molecule that can copy independently, approximately 16.5 KB in length, containing 37 genes that encode for 13 polypeptides involved in the electron transport chain (complexes I, III, IV, and V) responsible for oxidative phosphorylation, 22 tRNAs, and 2 rRNAs [95]. About 99% of mitochondrial proteins are synthesized on cytosolic ribosomes and then are introduced into mitochondria with specific proteins. Precursor protein translated by nuclear genes' mRNAs, without folding conformation, choose different mitochondrial membrane protein transfer enzyme complexes based on sequence information through the inside and outside mitochondria membrane. After entering the mitochondria, unfolded precursor proteins are folded by a molecular chaperone in the mitochondrial matrix [96]. Cells adopt various means to protect the mitochondrial proteome, including preventing the import of aberrant peptides, regulating protein turnover in mitochondria, and detecting protein homeostasis [97]. As an essential part of mitochondria, lipids' synthesis is similar to that of proteins: a small portion is made in mitochondria, and the rest is made in the endoplasmic reticulum before entering mitochondria [98].

### 5.1. Mitochondrial Biogenesis

The mtDNA is vulnerable to oxidative stress because of its proximity to the respiratory chain and lack of protective histone-like proteins and introns. Once mtDNA is damaged, encoding of critical proteins for the respiratory chain becomes deficient, aggravating ROS production and mitochondrial dysfunction. Moreover, mtDNA depletion alone could cause cardiomyocyte death. PGC-1, PPAR- (peroxisome proliferator-activated receptor-) *γ* coactivator-1, is a main factor regulating both mitophagy and mitochondrial biogenesis. The ectopic expression of PGC-1 in white adipose tissue upregulates transcription of UCP-1 and key enzymes of the mitochondrial respiratory chain and increases the cellular content of mtDNA. PGC-1 has two types: PGC-1*α* and PGC-1*β*. PGC-1*α* mainly regulates mitochondrial biogenesis by activating different transcription factors. PGC-1*α* activates nuclear respiratory factors 1 (NRF-1) and nuclear factor erythroid 2-related factor 2 (NRF2) to bind to NRF-related sites on the promoter of mitochondrial transcription factor A (Tfam) to increase its expression. NRFs regulate complexes' expression in the electron transport chain (ETC), while Tfam encoded by nuclear genes is responsible for transcription and replicating mtDNA [99]. Besides, PGC-1*α* interacts with and activates other transcription factors like PPARs and ERRs. ERR targets several genes linked to many biological metabolism processes and mitochondrial biogenesis [100]. Studies proved that AMPK, NO, SIRT1, and TORC1 control mitochondrial biogenesis by interacting with PGC-1*α* [101–104]. Yue et al. [105] found that IR injury decreased Tfam protein level and exposed mtDNA to oxidative damage, destroying the respiratory chain and overproducing ROS that could be reversed by antioxidant-like lycopene. S100A8/A9, the most significantly upregulated gene in the early reperfusion stage analyzed by dynamic transcriptome, downregulates NDUF gene expression through Toll-like receptor 4/ERK-mediated PPARG coactivator 1*α*/NRF 1 signaling, followed by mitochondrial complex I inhibition. This caused mitochondrial respiratory dysfunction in cardiomyocytes that can be reversed by S100a9 neutralizing antibody [106].

### 5.2. Mitochondrial Proteostasis

About 1,500 kinds of human mitochondrial proteins play a central role in cell energy metabolism and improve cell viability [107]. In addition to respiratory chain complexes involved in basic respiration, mitochondrial enzymes catalyze the biosynthesis of lipids and amino acids, central reactions of the urea cycle, and formation of heme and iron-sulfur clusters. Meanwhile, mitochondrial proteins could control cristae formation and maintenance, establish membrane contact sites with ER for lipid exchange, and regulate mitochondrial dynamics and signal transduction (such as calcium signaling) [108, 109]. The mitochondrial proteome shows high plasticity to allow the mitochondrial function to adapt to cellular requirements. Defects in mitochondrial protein homeostasis lead to toxic protein damage and ultimately cell death [110]. Thus, cell evolves several ways to maintain proteostasis.

Cytosolic protein quality control mechanism ensures that correctly synthesized polypeptide is imported and remains unfolded protein before being imported into mitochondria. The cytosolic ubiquitin-proteasome system (UPS) controls transmembrane transport of polypeptides and removes damaged and mislocalized protein. Chaperones in mitochondrial matrix help fold nuclear-encoded and mitochondrial-encoded protein properly. A selective autophagy approach independent of mitophagy can remove a portion or entire mitochondria via generating MDVs. Under mitochondrial oxidative stress conditions, ROS production triggers small vesicles that contain a subset group of oxidized proteins to bud off of damaged mitochondria to form MDVs. MDVs are fused with endosomes and multivesicular bodies and subsequently delivered to lysosomes to selectively degrade damaged mitochondrial contents [111]. In heart tissue, constant MDVs act as the first line of defense against stress in healthy conditions, and the number of MDVs is rapidly upregulated in response to stress [112]. A study has shown that MDVs inhibit the apoptosis of myocardial cells induced by hypoxia/ischemia through transferring Bcl-2 and play an endogenous protective role in early hypoxia [113]. However, the molecular mechanism controlling MDV formation remains unclear. ROS production also activates another mitochondrial remodeling and quality control mechanism, which is named mitochondrial spheroids. Mitochondrial spheroids have a ring or cup-like morphology with squeezed mitochondrial matrix and contain cytosol contents such as endoplasmic reticulum or other mitochondria. Forming mitochondrial spheroids is regulated by Mfn1 and Mfn2 and further acquires lysosomal markers to fusion with lysosomes [114]. Mitochondrial spheroids have been detected in mice's livers on acute alcohol or high-fat diets [115]. This suggests that mitochondrial spheroids may act as a general mitochondrial structural remodeling in response to various physiological and pathological stresses and may serve as a mechanism to regulate IR injury that needs to be further explored.

**Figure 2:**
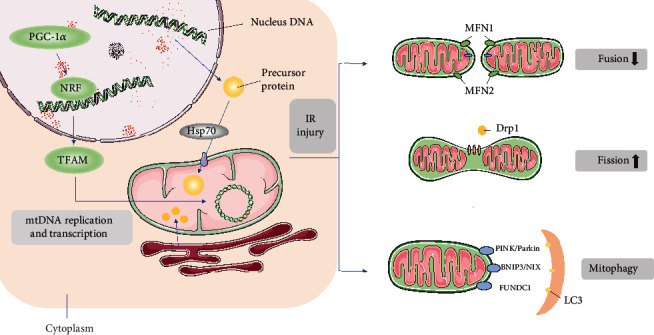
Quality control mechanism of mitochondria in IR injury. In normal mitochondrial biogenesis, mtDNA synthesis is mainly regulated by PGC-1, and PGC-1 interacts with nuclear receptors (including NRF-1 and NRF-2) to participate in the expression of various nuclear coding genes and Tfam, and transcription factors Tfam and NRFs are jointly responsible for regulating mtDNA replication and transcription. Nuclear DNA synthesizes precursor proteins in the cytoplasm and is transported in an unfolded form into the mitochondria, where precursor proteins fold into functional proteins with molecular chaperones. A small portion of lipids are synthesized in mitochondria, and the rest are transported into mitochondria after synthesis in the endoplasmic reticulum to form the inner and outer membrane structure. Mitochondrial fission is a signal of injury. After activation by phosphorylation, Drp1 translocates from the cytoplasm to the mitochondrial membrane and binds to Drp1 receptors (Mff, Fis1, and Mid49/51), which are the sites of enclosing mitochondria to be separated and mediate the mitochondrial division into fragments. Excessive mitochondrial fragmentation during IR eventually leads to cell death. Mitochondrial fusion inhibits mitochondrial fragmentation, reticular structure destruction, and mitochondrial cristae remodeling. Mfn1 and Mfn2 mediate OMM fusion, and Opa1 mediates IMM fusion. Mitochondrial fusion decreased significantly during IR injury. The function of mitophagy in myocardial IR remains unclear. Three main pathways are found to mediate mitophagy: PINK1/Parkin pathway may induce excessive mitophagy in myocardial IR, thereby promoting cell death; BNIP3/NIX is a protein located in OMM, which directly binds to LC3 on autophagosomes and mediates mitophagy. But its function in myocardial IR remains controversial; FUNDC1 is also an LC3 receptor located in mitochondria, and its LIR binds to LC3 to mediate mitophagy, which mainly plays a protective role in myocardial IR injury.

Besides, during stress events associated with abnormal mitochondrial protein accumulation, such as heat or oxidative stress, cells also launch UPR^mt^ through upregulating chaperones, proteases, and antioxidants to mitigate potential toxic protein damage [97, 116]. UPR^mt^ is regulated by transcription factor ATFS-1 in Caenorhabditis elegans and by transcription factor ATF5 in mammals. Under normal circumstances, ATF5 is imported to mitochondria and degraded by AAA^+^ proteases LON. When the cell is exposed to oxidative stress, the damaged protein homeostasis impedes importing ATF5 into mitochondria, resulting in ATF5 retention in the cytoplasm and subsequent translocation to the nucleus to enhance transcription of mitochondrial chaperones and proteases [117]. Interestingly, there are conflicting claims about the role of UPR^mt^ in heart disease. Most studies have shown that UPR^mt^ has a protective effect against heart injury, but a few studies have found that UPR^mt^ can promote heart disease development. Preinduction of UPR^mt^ with nicotinamide ribose is sufficient to prevent cardiac dysfunction caused by chronic hemodynamic overloading in rodents [118]. Increased and activation of UPR^mt^ was also found in the hearts of aging mice and cardiac tissue from patients with aortic stenosis [118, 119]. Under IR injury, pharmacological UPR^mt^ induction with oligomycin or doxycycline was cardioprotective in an ATF5-dependent manner in vivo. However, this approach did not reduce the severity of myocardial infarction in ATF5-deficient mice [120]. In addition, mammalian cells require ATF5 to maintain mitochondrial activity and promote organelle recovery during mitochondrial stress. These findings open a new avenue for cardiovascular disease treatment strategies targeting mitochondrial protein disorder under stress (Figure 2).

## 6. Interventions Based on Mitochondrial Quality Control

So far, among IR injury studies, the most likely mechanisms are overfission and abnormal mitophagy. However, the interaction between fission and mitophagy remains unclear. Mitochondrial fission is often a signal of cell damage. Overdivision induces mitochondrial fragmentation, aggravates oxidative stress, activates mitochondrial apoptosis, and reduces cell viability. In contrast, mitophagy is generally a protective signal. Normal mitophagy clears mitochondrial debris, protects oxidative stress, inhibits mitochondrial apoptosis, and maintains cell viability. However, abnormal fission and mitophagy will occur in IR injury, resulting in cell damage [121]. Given that myocardial cell is the status of terminal differentiation of cells after mitotic division, they cannot be further divided and subsequent cell replacement, therefore, removed by mitochondrial dysfunction of mitochondria balance between mitochondrial biology and health, mitochondria maintain steady is crucial to maintain a healthy heart and prevent cardiac injury [6]. We could consider mitochondrial quality control and intervene in any part of the overall quality control process to alleviate IR injury.

Several studies have currently improved IR damage by intervening in the quality control of mitochondria. Dapagliflozin administration before ischemia improves left ventricular function during cardiac IR injury by reducing myocardial apoptosis, improving myocardial mitochondrial function, biogenesis, and dynamics, thereby maximizing myocardial protection [122]. Various antioxidants such as melatonin can act as a protective factor against myocardial IR injury by regulating mitochondrial fission and mitophagy. Platelet activation is an important pathophysiological mechanism of IR injury. Melatonin can improve downregulation of PPAR*γ* expression after reperfusion, inhibiting platelet activation from attenuating myocardial IR injury by blocking FUNDC1-mediated mitophagy [123]. Underlying the microvascular IR injury, [Ca^2+^]i cannot be rapidly and timely recirculated into ER. [Ca^2+^]i accumulation not only leads to endothelial cell stiffness but also catalyzes XO to produce excessive ROS, which is the consequence of mitochondria damage. Sarcoplasmic/endoplasmic reticulum Ca^2+^-ATPase (SERCA) is a channel responsible for transporting [Ca^2+^]_i_ back to ER. It can reduce IR injury by inhibiting calcium overload, inactivating xanthine oxidase (XO), and reducing intracellular/mitochondrial ROS to regulate mitochondrial motility, bioenergy, biogenesis, and mitochondrial autophagy [124]. Istaroxime, as a nonglycoside inhibitor of sodium-potassium-ATPase, has an additional stimulatory effect on SERCA. Nitro donors have been developed and shown effective vasodilators in early animal studies [125]. Gene therapy by regulating circular RNAs is a promising means to improve heart contractile performance [126]. Combined SS31-mitochondria (Mito) therapy (rather than either therapy alone) increased SIRT1/SIRT3 expression and ATP levels, by which it suppressed oxidative stress and protected mitochondrial integrity. IR rats treated with SS31-Mito exhibited higher left ventricular ejection fraction (LVEF) and energy integrity (PGC-1*α*/mitochondrial cytochrome c) markers, demonstrating that combined SS31-Mito therapy is an efficient means to protect myocardium from IR injury [127]. Moreover, trimetazidine, electroacupuncture (EA) preconditioning also prevents IR injury by regulating mitochondrial quality and function [89]. Therefore, exploring the quality control mechanism that can regulate macroscopic mitochondria is highly demanding. However, when the number or overall state of mitochondria is not good, regulating the biogenesis of mitochondria itself or controlling unfolded proteins within mitochondria are also very worthy to be explored (Table 1).

## 7. Conclusion

Mitochondrial quality control has been well demonstrated as a central link in the IR injury mechanism mediated by Ca^2+^ overload and mPTP opening. Abnormal mitochondrial quality control, such as excessive fission and mitophagy, in addition to decreased mitochondrial fusion and proteostasis disorder, is a factor that further aggravates tissue damage. However, the extent to which mitochondrial clearance and production can damage tissue and save cell homeostasis remains unclear. The exact mechanism of the upstream regulatory pathway of mitochondrial quality control and factors that can affect mitochondrial function should be further investigated. The role of mitochondrial outer membrane proteins in IR injury is widely studied, but the proteins in mitochondria and interactions between mitochondria and other organelles such as ER perhaps play a similar important role in IR injury. Therefore, ensuring balance and interaction of portion of mitochondrial quality control seems critical to reducing damage under disease.

In addition, familiarity with physiological and pathological characteristics of mitochondrial quality control helps basic and clinical studies. Many studies have shown that genetic or pharmaceutical interventions in mitochondrial quality control can improve tissue damage and cardiovascular function caused by IR. But the limitation lies in that few studies have been clinically verified, most of which proved the efficacy of the drug in animal models. In conclusion, understanding the mechanism of mitochondrial action in IR damage can provide targeted therapeutic means for clinical use and develop new drug research. Furthermore, timely and effective intervention for the long-term pathological process of IR injury will significantly alleviate the degree of damage to the body to a certain extent.

## Figures and Tables

**Table 1:** Therapeutic application targeting MQC to attenuate IR injury.

Therapies	Mechanisms	References
Dapagliflozin	Improve left ventricular function	Lahnwong et al., 2020 [122]
Melatonin	Improve PPAR*γ* expression	Zhou et al., 2017 [123]
Istaroxime	Stimulate SERCA to dilate blood vessels and decrease ROS production	Hasenfuss et al., 2011 [125]
SS31-Mito	Enhance LVEF and energy integrity with higher PGC-1*α* and Cyt c	Lee et al., 2018 [127]

## References

[1] Tullio F., Angotti C., Perrelli M. G., Penna C., Pagliaro P. (2013). Redox balance and cardioprotection. *Basic Research in Cardiology*.

[2] Nicolás-Ávila J. A., Lechuga-Vieco A. V., Esteban-Martínez L. (2020). A network of macrophages supports mitochondrial homeostasis in the heart. *Cell*.

[3] Ni H. M., Williams J. A., Ding W. X. (2015). Mitochondrial dynamics and mitochondrial quality control. *Redox Biology*.

[4] Moehlman A. T., Youle R. J. (2020). Mitochondrial quality control and restraining innate immunity. *Annual Review of Cell and Developmental Biology*.

[5] Kulek A. R., Anzell A., Wider J. M., Sanderson T. H., Przyklenk K. (2020). Mitochondrial quality control: role in cardiac models of lethal ischemia-reperfusion injury. *Cell*.

[6] Tahrir F. G., Langford D., Amini S., Mohseni Ahooyi T., Khalili K. (2019). Mitochondrial quality control in cardiac cells: mechanisms and role in cardiac cell injury and disease. *Journal of Cellular Physiology*.

[7] Mokhtari-Zaer A., Marefati N., Atkin S. L., Butler A. E., Sahebkar A. (2018). The protective role of curcumin in myocardial ischemia-reperfusion injury. *Journal of Cellular Physiology*.

[8] Anzell A. R., Maizy R., Przyklenk K., Sanderson T. H. (2018). Mitochondrial quality control and disease: insights into ischemia-reperfusion injury. *Molecular Neurobiology*.

[9] Mittler R. (2017). ROS are good. *Trends in Plant Science*.

[10] Yang M., Linn B. S., Zhang Y., Ren J. (2019). Mitophagy and mitochondrial integrity in cardiac ischemia-reperfusion injury. *Biochimica et Biophysica Acta - Molecular Basis of Disease*.

[11] Ren J., Zhang Y. (2017). Editorial: new therapetic approaches in the management of ischemia reperfusion injury and cardiometabolic diseases: opportunities and challenges. *Current Drug Targets*.

[12] Zorov D. B., Juhaszova M., Yaniv Y., Nuss H. B., Wang S., Sollott S. J. (2009). Regulation and pharmacology of the mitochondrial permeability transition pore. *Cardiovascular Research*.

[13] Kwong J. Q., Molkentin J. D. (2015). Physiological and pathological roles of the mitochondrial permeability transition pore in the heart. *Cell Metabolism*.

[14] Crompton M., Costi A. (1990). A heart mitochondrial Ca2(+)-dependent pore of possible relevance to re-perfusion-induced injury. Evidence that ADP facilitates pore interconversion between the closed and open states. *The Biochemical Journal*.

[15] Han J. Y., Li Q., Ma Z. Z., Fan J. Y. (2017). Effects and mechanisms of compound Chinese medicine and major ingredients on microcirculatory dysfunction and organ injury induced by ischemia/reperfusion. *Pharmacology & Therapeutics*.

[16] He K., Yan L., Pan C. S. (2014). ROCK-dependent ATP5D modulation contributes to the protection of notoginsenoside NR1 against ischemia-reperfusion-induced myocardial injury. *American Journal of Physiology. Heart and Circulatory Physiology*.

[17] Pollard T. D., Borisy G. G. (2003). Cellular motility driven by assembly and disassembly of actin filaments. *Cell*.

[18] Harhaj N. S., Antonetti D. A. (2004). Regulation of tight junctions and loss of barrier function in pathophysiology. *The International Journal of Biochemistry & Cell Biology*.

[19] Ibanez B., James S., Agewall S. (2018). 2017 ESC guidelines for the management of acute myocardial infarction in patients presenting with ST-segment elevation: the task force for the management of acute myocardial infarction in patients presenting with ST-segment elevation of the European Society of Cardiology (ESC). *European Heart Journal*.

[20] Pell V. R., Chouchani E. T., Murphy M. P., Brookes P. S., Krieg T. (2016). Moving forwards by blocking back-flow. *Circulation Research*.

[21] Lefer A. M., Lefer D. J. (1996). The role of nitric oxide and cell adhesion molecules on the microcirculation in ischaemia-reperfusion. *Cardiovascular Research*.

[22] Bagheri F., Khori V., Alizadeh A. M., Khalighfard S., Khodayari S., Khodayari H. (2016). Reactive oxygen species-mediated cardiac-reperfusion injury: mechanisms and therapies. *Life Sciences*.

[23] Zhou H., Yang J., Xin T. (2014). Exendin-4 protects adipose-derived mesenchymal stem cells from apoptosis induced by hydrogen peroxide through the PI3K/Akt-Sfrp2 pathways. *Free Radical Biology & Medicine*.

[24] Zorov D. B., Filburn C. R., Klotz L. O., Zweier J. L., Sollott S. J. (2000). Reactive oxygen species (Ros-induced) ROS release. *The Journal of Experimental Medicine*.

[25] Kirichok Y., Krapivinsky G., Clapham D. E. (2004). The mitochondrial calcium uniporter is a highly selective ion channel. *Nature*.

[26] Boyman L., Karbowski M., Lederer W. J. (2020). Regulation of mitochondrial ATP production: Ca^2+^ signaling and quality control. *Trends in Molecular Medicine*.

[27] Kwong J. Q., Lu X., Correll R. N. (2015). The mitochondrial calcium uniporter selectively matches metabolic output to acute contractile stress in the heart. *Cell Reports*.

[28] Luongo T. S., Lambert J. P., Yuan A. (2015). The mitochondrial calcium uniporter matches energetic supply with cardiac workload during stress and modulates permeability transition. *Cell Reports*.

[29] Chan D. C. (2006). Dissecting mitochondrial fusion. *Developmental Cell*.

[30] Chen H., Detmer S. A., Ewald A. J., Griffin E. E., Fraser S. E., Chan D. C. (2003). Mitofusins Mfn1 and Mfn2 coordinately regulate mitochondrial fusion and are essential for embryonic development. *The Journal of Cell Biology*.

[31] Vásquez-Trincado C., García-Carvajal I., Pennanen C. (2016). Mitochondrial dynamics, mitophagy and cardiovascular disease. *The Journal of Physiology*.

[32] de Vos K. J., Allan V. J., Grierson A. J., Sheetz M. P. (2005). Mitochondrial function and actin regulate dynamin-related protein 1-dependent mitochondrial fission. *Current Biology*.

[33] Ingerman E., Perkins E. M., Marino M. (2005). Dnm1 forms spirals that are structurally tailored to fit mitochondria. *The Journal of Cell Biology*.

[34] Sharp W. W., Archer S. L. (2015). Mitochondrial dynamics in cardiovascular disease: fission and fusion foretell form and function. *Journal of Molecular Medicine (Berlin, Germany)*.

[35] Strack S., Wilson T. J., Cribbs J. T. (2013). Cyclin-dependent kinases regulate splice-specific targeting of dynamin-related protein 1 to microtubules. *The Journal of Cell Biology*.

[36] Wasiak S., Zunino R., Mcbride H. M. (2007). Bax/Bak promote sumoylation of DRP1 and its stable association with mitochondria during apoptotic cell death. *The Journal of Cell Biology*.

[37] Chang C. R., Blackstone C. (2007). Cyclic AMP-dependent Protein Kinase Phosphorylation of Drp1 Regulates Its GTPase Activity and Mitochondrial Morphology. *The Journal of Biological Chemistry*.

[38] Cereghetti G. M., Stangherlin A., de Brito O. M. (2008). Dephosphorylation by calcineurin regulates translocation of Drp1 to mitochondria. *Proceedings of the National Academy of Sciences of the United States of America*.

[39] Xu S., Wang P., Zhang H. (2016). CaMKII induces permeability transition through Drp1 phosphorylation during chronic *β*-AR stimulation. *Nature Communications*.

[40] Sharp W. W., Fang Y. H., Han M. (2014). Dynamin-related protein 1 (Drp1)-mediated diastolic dysfunction in myocardial ischemia-reperfusion injury: therapeutic benefits of Drp1 inhibition to reduce mitochondrial fission. *The FASEB Journal*.

[41] Suen D. F., Narendra D. P., Tanaka A., Manfredi G., Youle R. J. (2010). Parkin overexpression selects against a deleterious mtDNA mutation in heteroplasmic cybrid cells. *Proceedings of the National Academy of Sciences of the United States of America*.

[42] Brady N. R., Hamacher-Brady A., Gottlieb R. A. (2006). Proapoptotic BCL-2 family members and mitochondrial dysfunction during ischemia/reperfusion injury, a study employing cardiac HL-1 cells and GFP biosensors. *Biochimica et Biophysica Acta*.

[43] Karbowski M., Lee Y. J., Gaume B. (2002). Spatial and temporal association of Bax with mitochondrial fission sites, Drp1, and Mfn2 during apoptosis. *The Journal of Cell Biology*.

[44] Zhou H., Wang J., Zhu P. (2018). NR4A1 aggravates the cardiac microvascular ischemia reperfusion injury through suppressing FUNDC1-mediated mitophagy and promoting Mff-required mitochondrial fission by CK2*α*. *Basic Research in Cardiology*.

[45] Jin Q., Li R., Hu N. (2018). DUSP1 alleviates cardiac ischemia/reperfusion injury by suppressing the Mff- required mitochondrial fission and Bnip3-related mitophagy via the JNK pathways. *Redox Biology*.

[46] Zhou H., Hu S., Jin Q. (2017). Mff-dependent mitochondrial fission contributes to the pathogenesis of cardiac microvasculature ischemia/reperfusion injury via induction of mROS-mediated cardiolipin oxidation and HK2/VDAC1 disassociation-involved mPTP opening. *Journal of the American Heart Association*.

[47] Ono T., Isobe K., Nakada K., Hayashi J. I. (2001). Human cells are protected from mitochondrial dysfunction by complementation of DNA products in fused mitochondria. *Nature Genetics*.

[48] Ong S. B., Hausenloy D. J. (2010). Mitochondrial morphology and cardiovascular disease. *Cardiovascular Research*.

[49] Papanicolaou K. N., Kikuchi R., Ngoh G. A. (2012). Mitofusins 1 and 2 are essential for postnatal metabolic remodeling in heart. *Circulation Research*.

[50] Ishihara N., Eura Y., Mihara K. (2004). Mitofusin 1 and 2 play distinct roles in mitochondrial fusion reactions via GTPase activity. *Journal of Cell Science*.

[51] Hausenloy D. J., Yellon D. M. (2003). The mitochondrial permeability transition pore: its fundamental role in mediating cell death during ischaemia and reperfusion. *Journal of Molecular and Cellular Cardiology*.

[52] Ong S. B., Subrayan S., Lim S. Y., Yellon D. M., Davidson S. M., Hausenloy D. J. (2010). Inhibiting mitochondrial fission protects the heart against ischemia/reperfusion injury. *Circulation*.

[53] Griparic L., van der Wel N. N., Orozco I. J., Peters P. J., van der Bliek A. M. (2004). Loss of the Intermembrane Space Protein Mgm1/OPA1 Induces Swelling and Localized Constrictions along the Lengths of Mitochondria. *The Journal of Biological Chemistry*.

[54] Gegg M. E., Cooper J. M., Chau K. Y., Rojo M., Schapira A. H. V., Taanman J. W. (2010). Mitofusin 1 and mitofusin 2 are ubiquitinated in a PINK1/parkin-dependent manner upon induction of mitophagy. *Human Molecular Genetics*.

[55] Guan L., Che Z., Meng X. (2019). MCU up-regulation contributes to myocardial ischemia-reperfusion injury through calpain/OPA-1-mediated mitochondrial fusion/mitophagy inhibition. *Journal of Cellular and Molecular Medicine*.

[56] Zhang Y., Wang Y., Xu J. (2019). Melatonin attenuates myocardial ischemia-reperfusion injury via improving mitochondrial fusion/mitophagy and activating the AMPK-OPA1 signaling pathways. *Journal of Pineal Research*.

[57] Maneechote C., Palee S., Kerdphoo S., Jaiwongkam T., Chattipakorn S. C., Chattipakorn N. (2019). Balancing mitochondrial dynamics via increasing mitochondrial fusion attenuates infarct size and left ventricular dysfunction in rats with cardiac ischemia/reperfusion injury. *Clinical Science (London, England)*.

[58] Surinkaew P., Apaijai N., Sawaddiruk P. (2020). Mitochondrial fusion promoter alleviates brain damage in rats with cardiac ischemia/reperfusion injury. *Journal of Alzheimer's Disease*.

[59] Glick D., Barth S., Macleod K. F. (2010). Autophagy: cellular and molecular mechanisms. *The Journal of Pathology*.

[60] Yamamoto H., Kakuta S., Watanabe T. M. (2012). Atg9 vesicles are an important membrane source during early steps of autophagosome formation. *The Journal of Cell Biology*.

[61] Kim J., Kundu M., Viollet B., Guan K. L. (2011). AMPK and mTOR regulate autophagy through direct phosphorylation of Ulk1. *Nature Cell Biology*.

[62] Lu W., Karuppagounder S. S., Springer D. A. (2014). Genetic deficiency of the mitochondrial protein PGAM5 causes a Parkinson's-like movement disorder. *Nature Communications*.

[63] Meissner C., Lorenz H., Weihofen A., Selkoe D. J., Lemberg M. K. (2011). The mitochondrial intramembrane protease PARL cleaves human Pink1 to regulate Pink1 trafficking. *Journal of Neurochemistry*.

[64] Koyano F., Okatsu K., Kosako H. (2014). Ubiquitin is phosphorylated by PINK1 to activate parkin. *Nature*.

[65] Killackey S. A., Philpott D. J., Girardin S. E. (2020). Mitophagy pathways in health and disease. *The Journal of Cell Biology*.

[66] Yu L. M., Dong X., Xue X. D. (2021). Melatonin attenuates diabetic cardiomyopathy and reduces myocardial vulnerability to ischemia-reperfusion injury by improving mitochondrial quality control: role of SIRT6. *Journal of Pineal Research*.

[67] Zhou H., Zhang Y., Hu S. (2017). Melatonin protects cardiac microvasculature against ischemia/reperfusion injury via suppression of mitochondrial fission-VDAC1-HK2-mPTP-mitophagy axis. *Journal of Pineal Research*.

[68] Zhu Y., Massen S., Terenzio M. (2013). Modulation of Serines 17 and 24 in the LC3-interacting Region of Bnip3 Determines Pro-survival Mitophagy *versus* Apoptosis. *The Journal of Biological Chemistry*.

[69] Wang E. Y., Gang H., Aviv Y., Dhingra R., Margulets V., Kirshenbaum L. A. (2013). p53 mediates autophagy and cell death by a mechanism contingent on Bnip3. *Hypertension*.

[70] Sandoval H., Thiagarajan P., Dasgupta S. K. (2008). Essential role for Nix in autophagic maturation of erythroid cells. *Nature*.

[71] Zhang J., Ney P. A. (2009). Role of BNIP3 and NIX in cell death, autophagy, and mitophagy. *Cell Death and Differentiation*.

[72] Kubasiak L. A., Hernandez O. M., Bishopric N. H., Webster K. A. (2002). Hypoxia and acidosis activate cardiac myocyte death through the Bcl-2 family protein BNIP3. *Proceedings of the National Academy of Sciences of the United States of America*.

[73] Chinnadurai G., Vijayalingam S., Gibson S. B. (2008). BNIP3 subfamily BH3-only proteins: mitochondrial stress sensors in normal and pathological functions. *Oncogene*.

[74] Rikka S., Quinsay M. N., Thomas R. L. (2011). Bnip3 impairs mitochondrial bioenergetics and stimulates mitochondrial turnover. *Cell Death and Differentiation*.

[75] Regula K. M., Ens K., Kirshenbaum L. A. (2002). Inducible expression of BNIP3 provokes mitochondrial defects and hypoxia-mediated cell death of ventricular myocytes. *Circulation Research*.

[76] Chen G., Cizeau J., Vande Velde C. (1999). Nix and Nip3 form a subfamily of pro-apoptotic mitochondrial proteins. *The Journal of Biological Chemistry*.

[77] Diwan A., Krenz M., Syed F. M. (2007). Inhibition of ischemic cardiomyocyte apoptosis through targeted ablation of Bnip3 restrains postinfarction remodeling in mice. *The Journal of Clinical Investigation*.

[78] Li Y., Ren S., Xia J., Wei Y., Xi Y. (2020). EIF4A3-induced circ-BNIP3 aggravated hypoxia-induced injury of H9c2 cells by targeting miR-27a-3p/BNIP3. *Mol Ther Nucleic Acids*.

[79] Zheng X., Zhong T., Ma Y. (2020). Bnip3 mediates doxorubicin-induced cardiomyocyte pyroptosis via caspase-3/GSDME. *Life Sciences*.

[80] Hanna R. A., Quinsay M. N., Orogo A. M., Giang K., Rikka S., Gustafsson Å. B. (2012). Microtubule-associated protein 1 light chain 3 (LC3) interacts with Bnip3 protein to selectively remove endoplasmic reticulum and mitochondria via autophagy. *The Journal of Biological Chemistry*.

[81] Zhang Y., Liu D., Hu H., Zhang P., Xie R., Cui W. (2019). HIF-1*α*/BNIP3 signaling pathway-induced-autophagy plays protective role during myocardial ischemia-reperfusion injury. *Biomedicine & Pharmacotherapy*.

[82] Yang L., Xie P., Wu J. (2020). Deferoxamine treatment combined with sevoflurane postconditioning attenuates myocardial ischemia-reperfusion injury by restoring HIF-1/BNIP3-mediated mitochondrial autophagy in GK rats. *Frontiers in Pharmacology*.

[83] Zhu N., Li J., Li Y. (2020). Berberine protects against simulated ischemia/reperfusion injury-induced H9C2 cardiomyocytes apoptosis in vitro and myocardial ischemia/reperfusion-induced apoptosis in vivo by regulating the mitophagy-mediated HIF-1*α*/BNIP3 pathway. *Frontiers in Pharmacology*.

[84] Zhang W., Ren H., Xu C. (2016). Hypoxic mitophagy regulates mitochondrial quality and platelet activation and determines severity of I/R heart injury. *eLife*.

[85] Liu L., Feng D., Chen G. (2012). Mitochondrial outer-membrane protein FUNDC1 mediates hypoxia-induced mitophagy in mammalian cells. *Nature Cell Biology*.

[86] Wu W., Tian W., Hu Z. (2014). ULK1 translocates to mitochondria and phosphorylates FUNDC1 to regulate mitophagy. *EMBO Reports*.

[87] Zhou H., Zhu P., Wang J., Zhu H., Ren J., Chen Y. (2018). Pathogenesis of cardiac ischemia reperfusion injury is associated with CK2*α*-disturbed mitochondrial homeostasis via suppression of FUNDC1-related mitophagy. *Cell Death and Differentiation*.

[88] Chen G., Han Z., Feng D. (2014). A regulatory signaling loop comprising the PGAM5 phosphatase and CK2 controls receptor-mediated mitophagy. *Molecular Cell*.

[89] Xiao Y., Chen W., Zhong Z. (2020). Electroacupuncture preconditioning attenuates myocardial ischemia-reperfusion injury by inhibiting mitophagy mediated by the mTORC1-ULK1-FUNDC1 pathway. *Biomedicine & Pharmacotherapy*.

[90] Wu S., Lu Q., Wang Q. (2017). Binding of FUN14 domain containing 1 with inositol 1,4,5-trisphosphate receptor in mitochondria-associated endoplasmic reticulum membranes maintains mitochondrial dynamics and function in hearts in vivo. *Circulation*.

[91] Lim Y., Berry B., Viteri S. (2021). FNDC-1-mediated mitophagy and ATFS-1 coordinate to protect against hypoxia-reoxygenation. *Autophagy*.

[92] Yu W., Xu M., Zhang T., Zhang Q., Zou C. (2019). Mst1 promotes cardiac ischemia-reperfusion injury by inhibiting the ERK-CREB pathway and repressing FUNDC1-mediated mitophagy. *The Journal of Physiological Sciences*.

[93] Zhang W., Siraj S., Zhang R., Chen Q. (2017). Mitophagy receptor FUNDC1 regulates mitochondrial homeostasis and protects the heart from I/R injury. *Autophagy*.

[94] Baker M. J., Tatsuta T., Langer T. (2011). Quality control of mitochondrial proteostasis. *Cold Spring Harbor Perspectives in Biology*.

[95] Jornayvaz F. R., Shulman G. I. (2010). Regulation of mitochondrial biogenesis. *Essays in Biochemistry*.

[96] Baker M. J., Frazier A. E., Gulbis J. M., Ryan M. T. (2007). Mitochondrial protein-import machinery: correlating structure with function. *Trends in Cell Biology*.

[97] Song J., Herrmann J. M., Becker T. (2021). Quality control of the mitochondrial proteome. *Nature Reviews. Molecular Cell Biology*.

[98] Tatsuta T., Scharwey M., Langer T. (2014). Mitochondrial lipid trafficking. *Trends in Cell Biology*.

[99] Virbasius J. V., Scarpulla R. C. (1994). Activation of the human mitochondrial transcription factor A gene by nuclear respiratory factors: a potential regulatory link between nuclear and mitochondrial gene expression in organelle biogenesis. *Proceedings of the National Academy of Sciences of the United States of America*.

[100] Giguère V. (2008). Transcriptional control of energy homeostasis by the estrogen-related receptors. *Endocrine Reviews*.

[101] Zong H., Ren J. M., Young L. H. (2002). AMP kinase is required for mitochondrial biogenesis in skeletal muscle in response to chronic energy deprivation. *Proceedings of the National Academy of Sciences of the United States of America*.

[102] Nisoli E., Clementi E., Paolucci C. (2003). Mitochondrial biogenesis in mammals: the role of endogenous nitric oxide. *Science*.

[103] Rodgers J. T., Lerin C., Haas W., Gygi S. P., Spiegelman B. M., Puigserver P. (2005). Nutrient control of glucose homeostasis through a complex of PGC-1*α* and SIRT1. *Nature*.

[104] Wu Z., Huang X., Feng Y. (2006). Transducer of regulated CREB-binding proteins (TORCs) induce PGC-1*α* transcription and mitochondrial biogenesis in muscle cells. *Proceedings of the National Academy of Sciences of the United States of America*.

[105] Yue R., Xia X., Jiang J. (2015). Mitochondrial DNA oxidative damage contributes to cardiomyocyte ischemia/reperfusion-injury in rats: cardioprotective role of lycopene. *Journal of Cellular Physiology*.

[106] Li Y., Chen B., Yang X. (2019). S100a8/a9 signaling causes mitochondrial dysfunction and cardiomyocyte death in response to ischemic/reperfusion injury. *Circulation*.

[107] Morgenstern M., Stiller S. B., Lübbert P. (2017). Definition of a high-confidence mitochondrial proteome at quantitative scale. *Cell Reports*.

[108] Pfanner N., Warscheid B., Wiedemann N. (2019). Mitochondrial proteins: from biogenesis to functional networks. *Nature Reviews Molecular Cell Biology*.

[109] Giacomello M., Pyakurel A., Glytsou C., Scorrano L. (2020). The cell biology of mitochondrial membrane dynamics. *Nature Reviews Molecular Cell Biology*.

[110] Pickles S., Vigié P., Youle R. J. (2018). Mitophagy and quality control mechanisms in mitochondrial maintenance. *Current Biology*.

[111] McLelland G., Soubannier V., Chen C. X., McBride H., Fon E. A. (2014). Parkin and PINK1 function in a vesicular trafficking pathway regulating mitochondrial quality control. *The EMBO Journal*.

[112] Cadete V. J., Deschênes S., Cuillerier A. (2016). Formation of mitochondrial-derived vesicles is an active and physiologically relevant mitochondrial quality control process in the cardiac system. *The Journal of Physiology*.

[113] Li B., Zhao H., Wu Y. (2020). Mitochondrial-derived vesicles protect cardiomyocytes against hypoxic damage. *Frontiers in Cell and Development Biology*.

[114] Ding W. X., Guo F., Ni H. M. (2012). Parkin and mitofusins reciprocally regulate mitophagy and mitochondrial spheroid formation,. *The Journal of Biological Chemistry*.

[115] Ni H. M., Williams J. A., Jaeschke H., Ding W. X. (2013). Zonated induction of autophagy and mitochondrial spheroids limits acetaminophen-induced necrosis in the liver. *Redox Biology*.

[116] Wang J., Zhou H. (2020). Mitochondrial quality control mechanisms as molecular targets in cardiac ischemia - reperfusion injury. *Acta Pharmaceutica Sinica B*.

[117] Deng P., Haynes C. M. (2017). Mitochondrial dysfunction in cancer: potential roles of ATF5 and the mitochondrial UPR. *Seminars in Cancer Biology*.

[118] Smyrnias I., Gray S. P., Okonko D. O. (2019). Cardioprotective effect of the mitochondrial unfolded protein response during chronic pressure overload. *Journal of the American College of Cardiology*.

[119] Bozi L. H. M., Campos J. C., Gross E. R., Ferreira J. C. B. (2019). Mitochondrial Unfolded Protein Response (UPR^mt^) Activation in Cardiac Diseases: Opportunities and Challenges. *Journal of the American College of Cardiology*.

[120] Wang Y. T., Lim Y., McCall M. N. (2019). Cardioprotection by the mitochondrial unfolded protein response requires ATF5. *American Journal of Physiology-Heart and Circulatory Physiology*.

[121] Wong Y. C., Ysselstein D., Krainc D. (2018). Mitochondria-lysosome contacts regulate mitochondrial fission via RAB7 GTP hydrolysis. *Nature*.

[122] Lahnwong S., Palee S., Apaijai N. (2020). Acute dapagliflozin administration exerts cardioprotective effects in rats with cardiac ischemia/reperfusion injury. *Cardiovascular Diabetology*.

[123] Zhou H., Li D., Zhu P. (2017). Melatonin suppresses platelet activation and function against cardiac ischemia/reperfusion injury via PPAR*γ*/FUNDC1/mitophagy pathways. *Journal of Pineal Research*.

[124] Tan Y., Mui D., Toan S., Zhu P., Li R., Zhou H. (2020). SERCA overexpression improves mitochondrial quality control and attenuates cardiac microvascular ischemia-reperfusion injury. *Molecular Therapy - Nucleic Acids*.

[125] Hasenfuss G., Teerlink J. R. (2011). Cardiac inotropes: current agents and future directions. *European Heart Journal*.

[126] Zhang S., Wang W., Wu X., Zhou X. (2020). Regulatory roles of circular rnas in coronary artery disease. *Molecular Therapy - Nucleic Acids*.

[127] Lee F. Y., Shao P. L., Wallace C. G. (2018). Combined therapy with SS31 and mitochondria mitigates myocardial ischemia-reperfusion injury in rats. *International Journal of Molecular Sciences*.

